# Immune-modulatory effects of dietary Yeast Beta-1,3/1,6-D-glucan

**DOI:** 10.1186/1475-2891-13-38

**Published:** 2014-04-28

**Authors:** Heike Stier, Veronika Ebbeskotte, Joerg Gruenwald

**Affiliations:** 1 analyze & realize GmbH, Waldseeweg 6, 13467 Berlin, Germany; 2 Leiber GmbH, Hafenstraße 24, 49565 Bramsche, Germany

**Keywords:** Insoluble yeast β-glucans, Immune system, Clinical trial

## Abstract

Beta-glucans are a heterogeneous group of natural polysaccharides mostly investigated for their immunological effects. Due to the low systemic availability of oral preparations, it has been thought that only parenterally applied beta-glucans can modulate the immune system. However, several *in vivo* and *in vitro* investigations have revealed that orally applied beta-glucans also exert such effects. Various receptor interactions, explaining possible mode of actions, have been detected. The effects mainly depend on the source and structure of the beta-glucans. In the meantime, several human clinical trials with dietary insoluble yeast beta-glucans have been performed. The results confirm the previous findings of *in vivo* studies. The results of all studies taken together clearly indicate that oral intake of insoluble yeast beta-glucans is safe and has an immune strengthening effect.

## Introduction

A well-functioning immune system is crucial for staying healthy. Therefore, the potential of natural substances to strengthen the immune system has long been the subject of investigation. There are many synthetic and natural preparations claiming to be immunomodulators. Probably the best known herbal preparations that exhibit effects on the immune system are preparations made from *Echinacea, Viscum* (mistletoe), and *Pelargonium*. There is, however, another very interesting and, by now, properly investigated class of immunomodulators - the β-glucans.

Long before the substance class of β-glucans themselves were identified as immunomodulators, the beneficial effects of β-glucan-containing mushrooms such as Shiitake (*Lentinus edodes*) in Japan or Lingzhi (*Ganoderma lucidum*) in China were utilized in the traditional Oriental medicine for the strengthening of the body’s immune system.

The research on β-glucans started in the middle of the last century, when the immune-modulating effect of a yeast insoluble fraction was first shown [1]. Later it was demonstrated that the immunological activity of this preparation derives from the β-(1,3)-D-glucans [2].

Most of our current knowledge on the health benefits of β-glucans, the underlying mode of action, and its relationship to the structure of β-glucans was discovered within the last 20 years (for review see [3,4]).

Meanwhile, more than 6000 publications investigating the immune-modulating effects of β-glucans, such as anti-inflammatory or antimicrobial abilities, have been published [5]. Health effects were found not only in humans but also in invertebrates, rodents, fishes as well as farm animals such as cows or pigs (for review see [4]). Further, numerous studies reported other health benefits of β-glucans, including hepatoprotective, wound healing, weight loss, antidiabetic and cholesterol lowering functions (for review see [6,7]).

β-glucans are a heterogeneous group of natural polysaccharides, consisting of D-glucose monomers linked by a β-glycosidic bond. They are important structural elements of the cell wall or serve as energy storage in bacteria, fungi including yeast, algae, and plants, while they are absent in vertebrate and invertebrate tissue.

The individual glucose subunits are primarily linked either by (1,3)-β, (1,4)-β, or (1,6)-β glycosidic bonds. In most cases, β-glucans exhibit a uniformly constructed backbone of various lengths with side-chains of D-glucose attached by (1,4)-β, or (1,6)-β bindings.

However, not all β-glucans are able to modulate immune functions. These properties mainly depend on the primary chemical structure of the β-glucans. Cellulose for example, a (1,4)-β-linked glucan, does not exhibit immune-modulatory effects. In contrast, β-glucans derived from fungi and yeast, which consist of a (1,3)-β-linked backbone with small numbers of (1,6)-β-linked side chains, are essentially known for their immune-modulating effects [8].

Apart from differences in the type of linkage and branching, β-glucans can also vary in solubility, molecular mass, tertiary structure, degree of branching, polymer charge and solution conformation (triple or single helix or random coil). All these characteristics may influence their immune modulating effects [9]. On the other hand, the manufacturing process and, hence, the isolation method impacts the structure of β-glucans and consequentially their effects on the immune system. Indeed, the immune-modulating activity of different β-glucans from the same source might differ considerably in level of purity, solubility, molecular mass, tertiary structure, degree of branching, polymer charge and solution conformation [5].

Although there are numerous *in vitro* and *in vivo* investigations, human clinical trials confirming the preclinical findings are rather scarce. Due to different natural sources of the β-glucans, differences in application (intraperitoneal, intravenous or subcutaneous injections, or oral), differences in preparation and thus structural differences, the results obtained in the resulting clinical studies are non-homogeneous and often contradictory [4].

Due to this heterogeneity, we focused our review on the immune-modulating effects of insoluble yeast-derived dietary β-glucans.

The main focus will be on the food product Yestimun® - an insoluble, highly purified, well-characterized and intensively studied β-glucan from Spent Brewers’ Yeast. The *in vivo* data obtained from this preparation, together with the efficacy results obtained during clinical trials, will be presented and compared to the other dietary, insoluble yeast β-glucan preparations available on the market. Only randomized, controlled trials were considered for the purpose of this review. Possible mechanisms of action will be presented, based on the current knowledge about structure-function relationships of β-glucans. Studies investigating other aspects than the immune system or studies with soluble β-glucans were excluded.

## Characteristics of the proprietary yeast β-glucan preparation (Yestimun®)

Yestimun® is an insoluble (1,3)-(1,6)-β-glucan made from Spent Brewers’ Yeast (*Saccharomyces cerevisiae*). The brewers’ yeast used in Yestimun® is grown exclusively on malt and clean spring water with no other nutrients added. It is a natural by-product of the fermentation process used for beer production. Following gentle autolysis with the yeast’s own enzymes, high-performance centrifuges are used to separate the yeast autolysate into the soluble yeast extract and the insoluble yeast cell wall. Yeast cell walls contain typically about 30% of β-glucans of dry weight. During several separation processes, the β-glucans are further purified without the use of strong alkaline in the hydrolysis process, and soluble compounds are removed. Furthermore, the process does not involve an acidic hydrolysis, leaving the acid-sensitive β-1,6-glucan side chains mainly intact. This results in an average of 22% relative linkage percentage from β-1,6 glucan (H-NMR analysis according to FCC VII, 3rd suppl.; reference-glucan at 14%), with a minimum of purity of 85% (dry mass).

Growth conditions of the yeast might result in some heterogeneity of β-glucans within the cell wall [4]. Therefore, it can be speculated that β-glucans from cell walls of *S. cerevisiae* grown during brewery have a different β-glucan pattern than those from bakers’ yeast, which might also influence the immuno-modulating abilities of the product.

## Mode of action/structure function relationship

As humans cannot metabolize the β-glycosidic bonds from β-glucans, it has long been suspected that the bacterial fermentation process taking place within the intestinal system is involved in the health promoting effect of β-glucans.

Meanwhile, different possible mechanisms have been identified on how oral β-glucans modulate the immune system (for review see [10,11]).

In general, humans cannot synthesize β-glucans. Therefore, the immune system recognizes these compounds as foreign. The innate immune system responds to invading pathogens through pattern recognition receptors (PRR), which are typically expressed by immune cells but also by other cells. PRRs recognize conserved microbial structures, the so-called microbe-associated molecular patterns (MAMPs) [12], formally called PAMPs [13,14]. β-glucans are considered as one of the major MAMPs for the PRR-mediated sensing of fungal infection. So far, the most important PRRs for β-glucans are the dectin-1 receptor, the complement receptor 3 (CR3) and toll-like-receptors (TLR), which are found on various immune cells such as monocytes, macrophages, dendritic cells, neutrophils, eosinophils, and natural killer cells, but also on intestinal epithelial cells [10,15-17]. Binding of β-glucans to dectin-1 induced a cascade of innate and adaptive immune response such as phagocytosis, oxidative burst, and the production of cytokines and chemokines in dentritic cells and macrophages [15]. Kankkunen et al. showed that particulate yeast β-glucan triggered interleukin-1β (IL-1β) mediated cellular response in human primary macrophages via dectin-1 signaling [18]. Earlier *in vitro* studies showed that yeast β-glucan is a strong stimulant of macrophages [19] and induced mitogenic activity in rat thymocytes, indicating immunostimulatory effects [20].

The exact mechanism on how β-glucans affect immune function depends in part on the route of administration. Strong effects were already observed in studies in the early 1990s using intravenously injected yeast β-glucans [21-23], when the biological efficacy of orally administered β-glucans was critically discussed. In terms of oral administration, the impact on immune function is assumed to be primarily explained by the interaction of β-glucans with pinocytic microfold (M)-cells located in the small intestine [24]. It has been suggested that M-cells take up β-glucans and transport them from the intestinal lumen to the immune cells located within the Peyer’s patches [11].

Uptake of β-glucans has been shown in mice with soluble and particulate yeast (1,3)-(1,6)-glucans labeled with fluorescein. Both types of yeast β-1,3-glucans were taken up by gastrointestinal macrophages, and then processed forms were transported to the lymph nodes, spleen and bone marrow [25]. Also, *in vitro* experiments have shown that β-glucans were degraded inside macrophages and released into the culture medium [25], which makes them eventually available for the circulating system and a systemic distribution.

Orally administered β-glucans induced phagocytic activity, oxidative bursts, and IL-1 production of peritoneal macrophages in mice [26]. A higher phagocytic activity and oxidative metabolism of neutrophils and monocytes, indicating an immune restoring activity of yeast β-glucan has also been shown in rats [27]. However, not only the cellular but also the humoral acute phase immune reaction is affected by yeast β-glucan feeding as shown by increased lysozyme and ceruloplasmin activitiy [28].

Moreover, oral delivery of β-glucans impact mucosal immunity, as shown by an increase of intraepithelial lymphocytes in the intestine of mice [29]. In rats, the absorption of soluble β-glucans translocated from the gastrointestinal tract into the systemic circulation leads to an increased immune response and resistance against infectious challenge [16]. The effect of an insoluble β-glucan against anthrax infection in mice showed that the treated animals survived the anthrax infection, while 50% of the control animals died, indicating an improved immune function in animals fed with β-glucan [30].

Another important aspect to be considered is the solubility of β-glucans, as soluble and particulate (insoluble) β-glucans isolated from yeast may stimulate the immune system via different pathways [31]. *In vivo* and *in vitro* studies revealed that particulate (insoluble) β-glucan was phagocytosed by dendritic cells and macrophages via dectin-1 receptor pathway. Although particulate β-glucans can also be taken up by dendritic cells through a dectin-1 receptor independent mechanism, the dectin-1 receptor pathway is essential for the activation of dendritic cells, which in turn induces T-cell response and cytokine release [31]. However, not all insoluble particulate β-glucans are able to bind to and activate the dectin-1 receptor. Studies with synthetically produced β-glucans revealed that binding to dectin-1 receptor is specific for β-glucans with a (1,3)-beta backbone. Backbones with mixed (1,3)/(1,4)-β-bindings (e.g. barley derived β-glucans) are not recognized by this receptor. Also, a backbone length of at least seven glucose units is required for binding, in addition to one (1,6)-β-side-chain branch (e.g. insoluble yeast β-glucans). Furthermore, the binding activity increases with increasing molecular weight of the polymer [32]. However, binding to the dectin-1 receptor alone does not activate the signal cascade induced by this receptor. Indeed, dectin-1 signaling and the concomitant immune responses are only activated by particulate β-glucans but not by soluble β-glucans [33]. Insoluble, particulate β-glucans induced the process of phagocytosis, resulting in the elimination of invading microbes by binding to the dectin-1 receptor [31]. Further, particulate β-glucans promotes T-cell differentiation into Th1-cells and enhances cytotoxic T lymphocyte priming by the dectin-1 pathway. Even though soluble β-glucans are also recognized by the dectin-1 receptors, they cannot activate immune response via this pathway. Soluble β-glucans are, however, able to bind to the CR3 receptor. The activation of the CR3 leads to complement system mediated immune process, supported by specific antibodies [31].

These results show that different β-glucan particles influence the immune system via different pathways. Insoluble β-glucans are able to activate both the innate and the adaptive immune responses, whereas soluble β-glucans are most effective via the complement system, which needs specific antibodies.

## Studies performed with the proprietary yeast β-glucan preparation (Yestimun®)

### *In vivo* studies with Yestimun®

Feeding experiments with Yestimun® (previously called Biolex-Beta HP) in rats [34] showed that β-glucans increased the phagocytic activity of granulocytes and monocytes and the percentage of phagocytic cells. β-glucan feeding tended to have positive effects on the oxidative metabolism of these cell types. After stimulating monocytes with *E. coli,* the oxidative metabolism was significantly higher in the β-glucan group. Comparable effects were observed after phorbol myristate acetate (PMA) stimulation, a strong respiratory-burst stimulus [34].

Application of the same orally applied insoluble β-glucan resulted in an increase in non-specific humoral immune parameters in rats as shown by higher lysozyme and ceruloplasmin activities and serum γ-globulin levels [28]. This indicates that β-glucans may affect the synthesis of acute phase proteins. When the blood phagocytic cells were analyzed for their respiratory burst activity and their potential killing activity, the cells derived from the β-glucan fed group showed higher activity. Also, the proliferation rate of blood lymphocytes, when stimulated by Concanavalin A (ConA) or lipopolysaccharide (LPS), was higher in the β-glucan group, also indicating effects of β-glucans on cellular immunity [28].

These results were confirmed in another *in vivo* investigation in rats with cyclophosphamide suppressed immune systems [27]. Under these conditions, feeding β-glucans led to an increased phagocytic activity of monocytes and granulocytes. Also, the respiratory-burst activity and the oxidative metabolism of granulocytes and monocytes stimulated with formyl-Methionyl-Leucyl-Phenylalanine (fMLP), PMA and *E. coli* was increased [27].

In a very recent investigation, the same β-glucan preparation from Spent Brewers’ Yeast was, for 42 days, fed to dogs suffering from Inflammatory Bowel Disease (IBD) [35]. Within this time, the animals treated with β-glucan showed a significant improvement, measured by the Canine Inflammatory Bowel Disease Acitivity Index (CIBDAI). They further showed a decreased level of the pro-inflammatory IL-6 and an increase of the anti-inflammatory IL-10, as compared to untreated control animals [35].

From these experiments (see Table 1), it may be concluded that orally applied insoluble yeast β-glucans are able to strengthen a weakened immune system.

**Table 1:** ***In vivo *studies performed with the proprietary yeast β-glucan preparation Yestimun**^**®**^

**Title/reference**	**Study design/duration/dosage**	**Main results**
Effect of β-1,3/1,6-D-glucan on the phagocytic activity and oxidative metabolism of peripheral blood granulocytes and monocytes in rats [34]	20 adult rats were fed for 14 days with either 12–19 mg/rat/day β-1,3/1,6-D-glucan (Biolex-Beta HP*) or control diet	Arterial blood was analyzed for phagocytic activity and oxidative metabolism in blood granulocytes and monocytes:
		• significant higher phagocytic activity and a significant higher percentage of phagocytic cells in granulocytes and monocytes of β-glucan fed animal
		• positive effects on the oxidative metabolism of these cell types
		• monocytes stimulated by *E. coli* showed significantly higher oxidative metabolism in the β-glucan group
Effect of Biolex β-HP on selected parameters of specific and non-specific humoral and cellular immunity in rats [28]	20 adult rats were fed for 14 days with either 12–19 mg/rat/day β-1,3/1,6-D-glucan (Biolex-Beta HP*) or control diet	Orally applied insoluble β-glucans are able to increase non-specific humoral immune parameters in rats:
		• lysozyme and ceruloplasmin activities and serum gamma-globulin levels were significant higher
		• significant higher respiratory burst activity and potential killing activity of blood phagocytic cells
		• significant higher proliferation rate of blood lymphocytes when stimulated by ConA or LPS
Effect of Biolex Beta-HP on phagocytic activity and oxidative metabolism of peripheral blood granulocytes and monocytes in rats intoxicated by cyclophosphamide [27]	10 adult rats (control) and 10 rats treated with cyclophosphamide were fed for 14 days with β-(1,3)-(1,6)-D-glucan (Biolex-Beta HP*) at a dose of 50 mg/kg body weight/day or control diet	Under the condition of an experimentally suppressed immune system (treatment with cyclophosphamide), feeding with β-glucan led to:
		• increase of percentage of phagocytic monocytes and granulocytes
		• improvement of phagocytic activity of monocytes and granulocytes
		• a high level of oxidative metabolism of granulocytes and monocytes stimulated with fMLP, PMA and *E. coli*
The effectiveness of natural and synthetic immunomodulators in the treatment of inflammatory bowel disease in dogs [35]	28 dogs with IBD were treated for 42 days with	Feeding with β-glucan led to:
	I) β-(1,3)-(1,6)-D-glucan (Biolex-Beta HP*) at a dose of 7 mg/kg body weight/day	• decrease of the IBD symptoms measured by IBD Activity Index
	II) β-hydroxy- β-metyl butyrate 30 mg/kg bw/day	• decrease of the pro-inflammatory interleukin IL-6
	III) levamisol 2 mg/kg bw/day	• increase of the anti-inflammatory interleukin IL-10
	IV) control, without supplementation	

*Yestimun® was previously called Biolex-Beta HP.

### Clinical trials performed with the proprietary yeast β-glucan

Susceptibility to common cold is related to a weak immune status or a weak defense system. In the early 1990s, Cohen and colleagues demonstrated that the immune status of people with a high susceptibility to common colds is affected by lifestyle factors such as stress, emotional imbalance, mood, specific vitamin deficiencies, or exposure to wet conditions and low temperatures, and that the susceptibility is correlated to the occurrence of cold infections [36,37]. In contrast, a lower susceptibility to cold episodes reflects an improved defense against infections and, hence, a properly functioning immune system. Therefore, common cold is widely used as a proper model to investigate potential immune-modulating properties of natural substances, including β-glucans.

Two independent randomized, double-blind, placebo-controlled clinical trials showed that daily oral administration of the proprietary insoluble (1,3)-(1,6)-β-glucan, derived from brewers’ yeast, reduced the incidence of common cold episodes during the cold season in otherwise healthy subjects [38,39] (see Table 2).

**Table 2:** ***In vivo *studies performed with the proprietary yeast β-glucan preparation Yestimun**^**®**^

**Title/reference**	**Study design/duration/dosage**	**Main results**
Increased interleukin-10 but unchanged insulin sensitivity after 4 weeks of (1, 3)(1, 6)-beta-glucan consumption in overweight humans [40]	Randomized, double-blind, placebo-controlled, crossover study;	In overweight or obese subject the orally applied β-glucan leads to the following results:
	12 healthy overweight and obese subjects 3 × 0.5 g of (1,3)(1,6)-D-glucan (Biolex-Beta HP*) or placebo per day 2 × 4 weeks	• significant increase of both circulating levels and adipose tissue messenger RNA (mRNA) expression of the anti-inflammatory cytokine IL-10
		• insulin sensitivity was unaffected
		• no increase in non specific proinflammatory markers CRP, IL-6 and MCP-1/CCL-2
		• circulating levels and mRNA expression of proinflammatory cytokines were unaffected
A double-blind, randomized, placebo-controlled nutritional study using an insoluble yeast beta-glucan to improve the immune defense system [39]	Randomized, double-blind, placebo-controlled clinical trial;	Supplementation with 1,3/1,6-D-glucan led compared to placebo to:
	100 healthy adults with recurring common colds received 1,3/1,6-D-glucan (Yestimun®) 900 mg/day or a placebo for 26 weeks	• significant more subjects without incidences of common cold
		• significant less infections during the most intense infection season
		• significant reduction of the typical cold symptoms: “sore throat and/or difficulty swallowing”, “hoarseness and/or cough” and “runny nose”
Yeast (1,3)-(1,6)-beta-glucan helps to maintain the body’s defense against pathogens: a double-blind, randomized, placebo-controlled, multicentric study in healthy subjects [38]	Randomized, double-blind, placebo-controlled clinical trial;	Supplementation with 1,3/1,6-D-glucan led compared to placebo to:
	162 healthy adults with recurring common colds received 1,3/1,6-D-glucan (Yestimun®) 900 mg/day or a placebo for 16 weeks	• a significant reduced number (by 25%) of common cold infections in the per protocol population
		• a 15% lowered mean symptom score
		• significant reduced sleep difficulties usually caused by common cold episodes

*Yestimun® was previously called Biolex-Beta HP.

In the trial conducted by Graubaum et al. [39], 100 subjects received either the yeast-derived β-glucan preparation (900 mg/day) or a placebo over a period of 26 weeks. Results showed that, compared to placebo, more subjects in the intervention group did not have a common cold infection at all. The β-glucan group had significantly more subjects without a common cold episode than the placebo group. During the most intense infection season, the β-glucan group had significantly less infections compared to placebo. Ingestion of β-glucans significantly reduced the typical cold symptoms [39].

The second trial with 162 participants confirmed these results, as the number of cold episodes was reduced by 25% in the β-glucan group, compared to the placebo group (p = 0.041) [38]. Moreover, the authors reported a milder progression of severe common cold episodes in subjects supplemented with β-glucans. Also, sleeping difficulties, regarded as a side effect of a cold infection, were improved by the supplementation of β-glucan (p = 0.028).

The results of the common cold studies in healthy adults showed the immune-stimulating effects of the same brewers’ yeast β-glucan preparation. As an immunomodulator, however, β-glucans are also able to induce anti-inflammatory abilities. Kohl et al. [40] showed an anti-inflammatory action of the same β-glucan preparation in overweight and obese subjects. Obesity is often associated with inflammatory conditions that lead to an activation of the innate immune system. Long-term activation of the immune system may cause several other health related problems, including insulin resistance [40] (see Table 2). Indeed, yeast β-glucan consumption had an impact on immune function, as shown by an increase of both circulating levels and adipose tissue messenger RNA (mRNA) expression of the anti-inflammatory cytokine IL-10. Insulin sensitivity as well as circulating levels and mRNA expression of pro-inflammatory cytokines were, however, unaffected. The results indicate that intake of particulated yeast β-glucans also has anti-inflammatory properties.

These studies provide evidence on the potential immunmodulatory effects of yeast β-glucans: On one hand, the substances elicit/amplify (activate) the immune reaction as shown in the prevention of infections; on the other hand, they are capable of reducing the inflammatory reaction by inducing anti-inflammatory processes.

The clinical findings confirmed the previous results of the *in vivo* investigations performed with Yestimun® investigating the immunomodulatory effect (see Table 1).

Altogether, these *in vivo* studies, together with the human clinical trials, provide evidence on the substantial biological relevance of the β-glucan preparation Yestimun® on the immune system.

### Clinical trials with other dietary yeast β-glucans

A search for other placebo-controlled human trials performed with β-glucans derived from yeast (*Saccharomyces*) in the field of respiratory tract infection or common cold was performed in PubMed database. The main search terms were “controlled clinical trial” AND, “common cold OR respiratory tract infection” in combination with “β-glucan AND yeast”. Apart from the clinical trials performed with Yestimun®, we found nine other studies that fit our search criteria [22,41-48]. The publications by Babineau et al. [21] and Dellinger et al. [41] will not be discussed further, since the β-glucans were applied intravenously and not orally, to a group of high-risk surgical patients.

The following human studies were all performed with the same insoluble bakers’ yeast β-glucan preparation Wellmun WGP® (relative% of 1,6 linkage: 10%-18%; H-NMR analysis according to FCC VII, 3rd suppl.; purity: minimum of 75% of dry matter). In one intervention, healthy community-dwelling subjects (n = 40) received either an insoluble β-glucan preparation (500 mg/day) or placebo. Intake of β-glucans did not result in any significant difference in incidences of respiratory infections. Even though the number of infections was not different, none of the subjects supplemented with β-glucans missed school or work due to colds as opposed to placebo [42].

Similar results were obtained when healthy individuals (n = 79) received either 250 mg/day β-glucans or placebo over a period of 90 days during the peak upper respiratory tract infection (URTI) season. Even though there was no statistically significant difference in the total number of days with reported URTIs (β-glucans 198 days in 4.6%, versus 241 days in 5.5% in the control group, p = 0.06), the symptoms tended to be lesser in the β-glucan group. Of all assessed symptoms, only the item “ability to breathe easily” was significantly better in the β-glucan group than in the placebo group [47].

The ability to minimize post-exercise-induced immune suppression was measured in physically active subjects (n = 60), who consumed either 250 mg insoluble β-glucans or placebo (rice flour; cross-over design) for 10 days (pre-exercise supplementation period) before a bout of cycling. Blood analysis showed that ingestion of β-glucans significantly increases the total number of monocytes as well as of pro-inflammatory monocytes (p < 0.05). Further *ex vivo* LPS stimulation significantly increased plasma cytokine production of IL-2, Il-4, IL-5, and interferon-gamma [46].

These *ex vivo* results confirmed the results obtained from marathon runners (n = 75), who randomly received either 250 or 500 mg/day β-glucan or a placebo over four weeks post-marathon [43]. Particulate β-glucans significantly reduced URTI symptoms compared to placebo (p < 0.05). The number of URTI after four weeks post-marathon in both β-glucan groups was reported to be only 8% compared to 24% in the placebo group (not significant).

Long-term stress is another factor known to weaken the immune system. When stressed women (n = 77) took insoluble bakers’ yeast β-glucan before breakfast for 12 weeks, they reported fewer upper respiratory tract symptoms compared to placebo (p < 0.05) and a better overall well-being (p < 0.05) [48]. Similar results have been obtained in moderate to highly stressed subjects (n = 150; placebo n = 50; 250 mg β-glucan/day n = 50; 500 mg β-glucan/day n = 50) [44] as well as healthy stressed subjects (screened for moderate level of psychological stress n = 122) [45]. Once again, the subjects reported significantly fewer URTI symptoms and improved well-being (p < 0.05).

Although most of the trials mentioned above were underpowered and the differences in number of infections were not always significant compared to placebo, all are in support of the positive effects of insoluble β-glucans on the human immune system. The differences in the studies conducted with different β-glucan preparations may be explained by varying study conditions, sample size, study populations, applied dosages, and the different sources or isolation methods of β-glucan from brewers’ yeast or bakers’ yeast.

These clinical trials were all performed with insoluble yeast β-glucans. Only one human clinical trial investigating the immune effect of dietary soluble yeast β-glucans has been found [49]. In that pilot trial, dietary intake of soluble β-glucans leads to an increase in salvia IgA concentration. Based on this pilot trial it is not possible to come to a final conclusion that also soluble yeast β-glucans, when applied orally, are able to strengthen the immune system the way it was shown for the insoluble yeast β-glucan fraction.

Even though many of the clinical trials performed with insoluble β-glucan preparations showed positive effects on the immune system, none of them were so far able to convince the European Food Safety Authority (EFSA) to accept a health claim application in the area immune system. EFSA rejected all generic claim applications (13.1 claims) regarding beta-glucan and immune function. Also, the two product-specific 13.5 health claims submitted for the product Yestimun® were rejected [50,51]. The Panel concluded that a cause and effect relationship has not been established. The major reasons for rejection in those cases were the use of a non-validated questionnaire on common cold as well as limitations of the statistical analysis. However, EFSA has not granted any of the applications for product-specific 13.5 health claims on immune function. Only generic 13.1 claims for vitamins and minerals have been accepted [52,53]. These numerous rejections indicate the very high benchmark demanded by EFSA for claim substantiation on immune function.

## Safety

Brewers’ and bakers’ yeast (*S. cerevisiae*) has a long history of safe consumption. It has been used for over a thousand of years in the production of bread, wine, and beer. Although allergies to *S. cerevisiae* from food consumption might occur [54], they are rare. In general, yeast products are very well tolerated. However, no studies addressing the beneficial as well as adverse effects due to long-term intake of β-glucans are available as yet. In a scientific opinion, the EFSA Panel on Dietetic Products, Nutrition and Allergies (NDA) considered “that the allergenic risk of the ‘yeast β-glucans’ is not higher than the risk from other products containing bakers’ yeast. β-glucans from other sources have already been evaluated for safety by EFSA” [55]. The result of the EFSA evaluation is, among others, based on the significant history of safe use of yeast and on data from human and animal studies.

In the above-presented human studies with orally applied yeast β-glucans, no signs of toxicity were reported. In all these clinical trials, insoluble yeast β-glucans were very well tolerated. When adverse events occurred, they were either not associated with the intake of the study products, or they were comparable to the intake of placebo.

Single-dose acute and sub-chronic toxicity studies in rats did not result in toxic effects of insoluble bakers’ yeast (*S. cerevisiae*) preparations. Single dosages of 2000 mg/kg did not lead to death or clinical abnormalities in any of the tested rats. Also, oral application over 91 days of up to 100 mg/kg body weight did not lead to adverse or toxic effects in rats [56].

In some publications, insoluble β-glucans are stated to exhibit undesirable toxicological effects such as hepatosplenomegaly and granuloma formation. However, these adverse reactions were only associated with intravenous application [57,58]. Such adverse events never have been reported after oral application.

The safety of β-glucans can also be deduced from the mode of action. β-glucans do not directly attack the infected cells or the infection causing agents, but instead modulate the host’s defense mechanism. For example, macrophages were activated, but only reacted if foreign cells (bacteria, viruses, parasites) invade the system.

## Conclusion

Many investigations on the immunomodulatory effects of β-glucans were performed using parenteral applications. In the meantime, those effects have also been shown for orally applied β-glucans. Therefore immune stimulating effects may be achieved by dietary intake of yeast β-glucans. Despite those positive effects the European Food Safety Authority did not accept health claim applications of β-glucans in the area immune system. Based on several weaknesses regarding study design and statistical evaluations the panel concluded that a cause and effect relationship has not been established.

β-glucans from yeast are recognized by immune cells within the intestinal mucosa, amongst others by the dectin-1 receptor. Dectin-1 receptor activation induces several immune-stimulating effects important in the defense against invading pathogens. Furthermore, following uptake of β-glucans via dectin-1-stimulated phagocytosis, degradation processes within macrophages may make β-glucans systemically available.

However, not all β-glucan preparations have the potential to stimulate these reactions. In order to be able to activate the dectin-1 receptor cascade, β-glucans must comply with specific structural properties. It seems that insoluble, particulate (1,3)-β-glucans with 1,6-β-branches are able to activate this cascade, while soluble ones activate the antibody-mediated complement system via the CR3 receptor.

Clinical trials performed with dietary insoluble particulate β-glucans have demonstrated positive effects on the immune system. Therapeutic efficacy results for orally applied soluble yeast β-glucans are not yet available. Whether or not the soluble fraction of the yeast β-glucan, when orally applied, is equally active when influencing the human immune system needs to be proven in confirmative clinical trials. So far, this has only been shown for the insoluble fraction.

All the performed human clinical trials demonstrated that intake of β-glucans isolated from brewers’ yeast is very well tolerated. Based on the clinical trials presented in this review, an increased intake of dietary β-glucans might help to improve immune functions.

## Competing interests

This work has been funded by Leiber GmbH (Bramsche, Germany), the manufacturer of the yeast β-glucan preparation Yestimun®. VE is an employee of Leiber GmbH; HS and JG are employees of the contract research organization (CRO) analyze & realize GmbH, and where previously involved in the publication of two clinical trials with Yestimun®.

## Authors’ contributions

HS has drafted and written the manuscript. VE and JG have been involved in drafting the manuscript and revising it critically for important intellectual content. All authors read and approved the final manuscript.

## Authors’ information

HS is working as a Scientific Consultant for the CRO analyze & realize GmbH (Germany); JG is the founder of the CRO analyze & realize GmbH (Germany). VE is working as a scientist in the technical service of the animal nutrition department of Leiber GmbH (Bramsche, Germany).
